# Angular Deformities of the Knee in Children Treated with Guided Growth

**DOI:** 10.5704/MOJ.2107.005

**Published:** 2021-07

**Authors:** K Jamil, MY Yahaya, AF Abd-Rasid, S Ibrahim, AH Abdul-Rashid

**Affiliations:** 1 Department of Orthopaedics and Traumatology, Universiti Kebangsaan Malaysia, Kuala Lumpur, Malaysia; 2 Department of Orthopaedics, Universiti Teknologi Mara, Batu Caves, Malaysia; 3 Faculty of Medicine, Universiti Teknologi Mara, Sungai Buloh, Malaysia

**Keywords:** bone plate, genu valgum, genu varum, growth modulation, growth plate

## Abstract

**Introduction:**

The guided growth technique is an alternative to corrective osteotomy for treating angular deformities of the extremities. It has the advantage of being minimally invasive and is effective in a growing child. This study reports on the outcome of guided growth technique using a plate in correcting knee angular deformities.

**Material and Methods:**

We conducted a retrospective study of children with angular deformity of the knee treated by the guided growth technique from January 2010 to December 2015 in a tertiary centre. The guided growth technique was done using either the flexible titanium plate (8-plate) or the 2-hole reconstruction plate. Correction of deformity was assessed on radiographs by evaluating the mechanical axis deviation and tibiofemoral angle. The implants were removed once deformity correction was achieved.

**Results:**

A total of 17 patients (27 knees) were evaluated. Twenty-two knees (81.5%) achieved complete correction of the deformity. The median age was 4.0 (interquartile range 3.0-6.0) years and the median Body Mass Index (BMI) was 26.0 (25.0-28.0). There were 7 unilateral and 10 bilateral deformities with different pathologies (14 tibia vara, 3 genu valgus). The median rate of correction was 0.71° per month. One patient (1 knee) had screw pull-out and two patients (4 knees) had broken screws in the proximal tibia. Three patients (5 knees) failed to achieve complete correction and were subsequently treated with corrective osteotomies. Out of five patients (8 knees) who were followed-up for at least 12 months after removal of hardware, two had rebound deformities. No permanent growth retardation occurred in our patients.

**Conclusion:**

Our outcome for guided growth to correct knee angular deformity was similar to other studies. Guided growth is safe to perform in children below 12 years old and has good outcome in idiopathic genu valgus and Langeskiold II for tibia vara. Patients should be observed for recurrence until skeletal maturity following implant removal.

## Introduction

Angular deformities of the lower limbs may cause abnormal gait, ligamentous laxity, abnormal loading and potentially accelerate the degenerative changes of the knee^1-3^. When surgical correction is indicated, corrective osteotomy is one of the options of treatment. However, this procedure is not without complications. Post-operative site morbidity, pain and restricted weight-bearing leading to long absence from school are the main drawbacks of this surgery^4^. Gradual correction with external fixation is another reliable method for angular deformity correction, but has many disadvantages which include low patient compliance, high complication rates and long duration of treatment^2^.

In children, physeal manipulation with less invasive surgeries for correcting angular deformity, has a smaller risk of morbidity. Examples of these procedures include physeal bridge resection (epiphysiolysis), physeal distraction (chondrodiastasis), and partial growth arrest (hemiepiphysiodesis), which can be done as a temporary or permanent procedure. The least invasive of these options is partial growth arrest (hemiepiphysiodesis). This technique involves creating a tether across the convex side of the angular deformity and follows the Hueter-Volkmann law, whereby a compressive force across the physis results in a growth arrest at the treated part^2^. The procedure also relies on the growth potential of the opposite unarrested side of the physis. While one side of the growth is slowed down, the opposite side will continue to grow with gradual correction of the angulation.

Temporary hemiepiphysiodesis is a method that is increasingly being used for angular deformity correction in children and adolescents^5^, as it allows continuation of normal growth following the procedure. There are several types of temporary hemiepiphysodesis techniques. Percutaneous epiphysiodesis using transphyseal screws is quick and simple, but there is a concern of irreversible and complete growth arrest because it traverses the physeal plate^6^. Stapling is another widely used method but has reported complication of extrusion of staples^3,7^. Guided growth technique using the plate system is also a viable option, and reportedly has less complications than stapling^8,9^. This study reports the outcome of using flexible titanium plates (8-plates) and reconstruction plates for temporary hemiepiphysiodesis in correcting angular deformities of the knee in children.

## Materials and Methods

Following the institutional ethics committee approval, all children with angular deformity of the knee treated by guided growth using either an 8-plate or reconstruction plate at our centre from January 2010 to December 2015 were included in the study. Patients without a full-length radiograph done pre-operatively and after full correction were excluded. The patients’ information and demographic data were obtained from clinical records. Baseline data such as the Body Mass Index (BMI), sides of knee affected, types of angular deformity (varus or valgus) and their underlying causes were recorded.

**Fig. 1:**
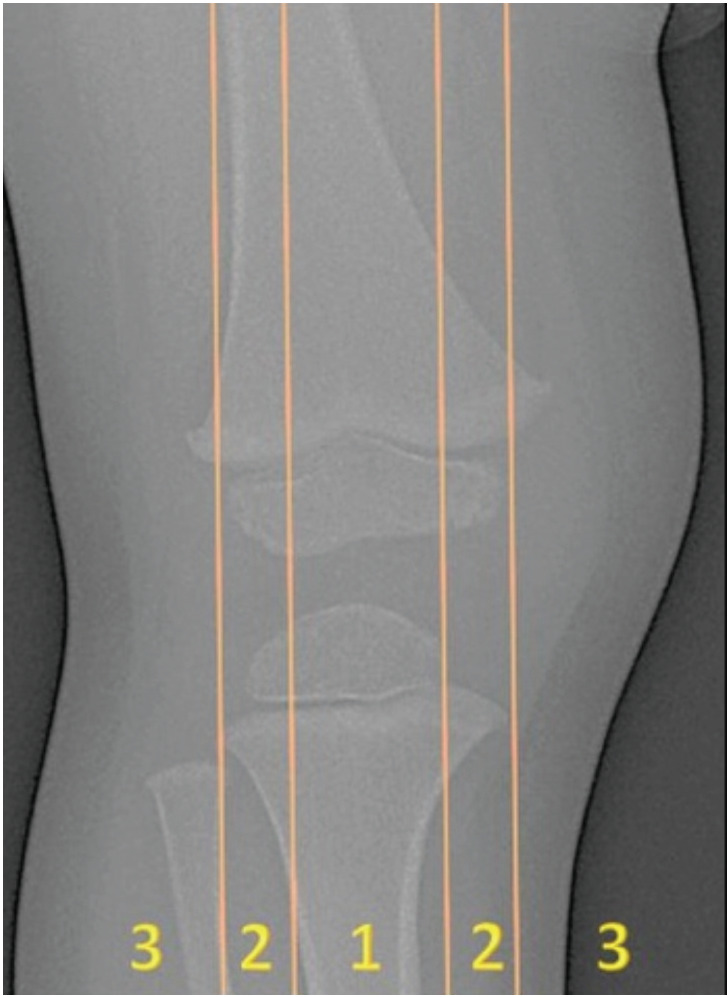
The three zones of the knee on the anteroposterior radiograph. Mechanical axis drawn from the centre of the femoral head to the centre of the ankle joint should pass through the central part of the knee joint (zone 1). Zones 2 and 3 lie outside the mechanical axis.

The surgical indications were pathological varus or valgus deformities of the knee. These involved children aged three years and above with tibiofemoral angle (TFA) of 15° or more, which did not improve or worsened after six months of observation. As described by Stevens *et al*^10^, we define excessive deformities when there was displacement of mechanical axis (MAD) of lower extremities to zone 2 or zone 3 in the knee joint on standing full-length radiograph of the lower limbs (Fig. 1). The mechanical axis is a line connecting the centre of femoral head to the centre of ankle joint. The deformity was then further assessed by measuring the TFA on the full-length radiograph of the lower extremity. The surgical procedure had been described by Stevens^11^. Intra-operative C-arm was used to identify the physis of the intended operative site in all cases. Through a 2cm to 3cm incision, the plate was placed submuscularly, superficial to the preserved periosteum. We used either the flexible titanium plate system; 8-plate [Orthofix, McKinney, TX, USA] or the non-cannulated screw reconstruction plate system; recon plate [Synthes, Davos, Switzerland]. When the reconstruction plate was used, a 4-hole plate was cut into two and contoured to the shape of the bone. The 8-plate has its own guide wire and cannulated screws. The procedure was performed on either the lateral or the medial side of the knee depending on the apex of the deformity without contouring the plate. Screw diameter of 3.5mm was inserted parallel to the physis with the length selected to reach at least midline of the epiphysis. Post-operatively, the patients were allowed to weight bear as soon as the pain was tolerable. They were followed-up every four months with radiographic assessment until complete correction of the deformity was achieved. The patients were then advised to come for a six-monthly appointment following implant removal to assess for recurrence of deformity.

Correction of angular deformity was determined by the value of mechanical axis deviation (MAD) and the measurements of the tibio-femoral angle (TFA) on standing full-length radiograph of the lower extremity. The final outcome was evaluated as resolved (when the MAD remains in zone 1 of the knee joint) or failed (MAD uncorrected). Once resolved, implant removals were planned on the next available theatre list. The amount of correction achieved was determined by the difference of the TFA measured on the pre-operative and the post-operative radiographs, before removal of implant. Rate of correction was calculated by the amount of correction (in degrees) divided by the duration needed for complete correction (in months). Rebound deformity was defined when the corrected mechanical axis has deviated back from zone 1 to either zones 2 or 3 at the knee joint.

Fisher exact test for comparison of categorical variables and Mann-Whitney U test for continuous data were utilised. Spearman rho test was used for correlations. Statistical analysis was performed using SPSS [v24, IBM, NY, USA] where statistical significance was assumed for p < 0.05.

## Results

All the 17 children were followed-up at least until implant removal, but only five children had 12 months or more follow-up after plate removal. There were 12 males (18 knees) and 5 females (9 knees). Results are presented as median and interquartile range. The median age was 4.0 years old (3.0 to 6.0 years). The median BMI was 26.0 (25.0-28.0). There were 7 unilateral and 10 bilateral deformities with varying pathologies. The causes of the deformities were tibia vara (Blount disease) (n=14), idiopathic genu valgus (n=2) and Down syndrome with bilateral genu valgus (n=1). Table I shows the patients’ demographics and data characteristics.

**Table I:** Demography and characteristics. F=Female; M=Male; Rt=right; Lt=left; B=bilateral; U=unilateral; NA=not applicable

Case	Age (years)	Sex	BMI	Side	Diagnosis	Langeskiold stage	Pre-operative TFA (degrees)	Pre-removal of implant TFA (degrees)	Duration of implant (months)	Correction rate (degrees/ month)	Age at Implant removal (years)	Age at last follow-up removal (years)	Rebound	Implant	Outcome
1	6	F	22	B	Down syndrome with genu valgus	NA	Rt 20 , Lt 20	Rt 6, Lt 6	Rt 23, Lt 23	Rt 0.61, Lt 0.61	Rt 8, Lt 8	8	No	8-plate	Resolved
2	3	M	22	U	Idiopathic genu valgus	NA	16	8	11	0.73	4	10	No	Reconstruction plate	Resolved
3	4	M	20	U	Idiopathic genu valgus	NA	24	8	10	1.60	5	5	No	8-plate	Resolved
4	3	M	25	U	Tibia vara	II	30	2	14	2.00	4	4	No	8-plate	Resolved
5	6	M	26	U	Tibia vara	II	24	5	10	1.90	7	7	No	8-plate	Resolved
6	4	M	28	U	Tibia vara	II	26	5	13	1.62	5	5	No	8-plate	Resolved
7	4	M	28	U	Tibia vara	II	15	6	20	0.45	6	6	No	8-plate	Resolved
8	3	M	25	B	Tibia vara	II	Rt 37, Lt 37	Rt 5, Lt 1	21	Rt 1.52, Lt 1.71	Rt 5, Lt 5	6	Yes	8-plate	Resolved
9	13	M	32	B	Tibia vara	NA (adolescent)	Rt 30, Lt 20	Rt 30, Lt 20	33	0	Failed	Failed	No	8-plate	Failed
10	3	F	27	B	Tibia vara	III	Rt 18 , Lt 20	Rt 5, Lt 6	Rt 27, Lt 38	Rt 0.48, Lt 0.37	Rt 5, Lt 6	6	No	8-plate	Resolved
11	9	F	25	B	Tibia vara	III	Rt 18 , Lt 18	Rt 7, Lt 6	Rt 17, Lt 17	Rt 0.65, Lt 0.71	Rt 10, Lt 10	16	No	8-plate	Resolved
12	4	M	28	B	Tibia vara	II	Rt 15 , Lt 18	Rt 6, Lt 7	Rt 31, Lt 44	Rt 0.29, Lt 0.25	Rt 6, Lt 7	8	No	8-plate	Resolved
13	6	F	26	B	Tibia vara	III	Rt 20 , Lt 20	Rt 5, Lt 15	Rt 21, Lt 21	Rt 0.71, Lt 0.29	Rt 8, Lt Failed	13	Yes	8-plate	Rt rebound Lt Failed
14	7	M	28	B	Tibia vara	II	Rt 15 , Lt 15	Rt 5, Lt 5	Rt 39, Lt 25	Rt 0.26, Lt 0.40	Rt 10, Lt 9	10	No	8-plate	Resolved
15	4	F	28	U	Tibia vara	II	17	7	11	0.91	5	5	No	8-plate	Resolved
16	5	M	26	B	Tibia vara	II	Rt 20 , Lt 20	Rt 5, Lt 5	Rt 16, Lt 16	Rt 0.94, Lt 0.94	Rt 6, Lt 6	6	No	8-plate	Resolved
17	3	M	28	B	Tibia vara	III	Rt 25 , Lt 20	Rt 19, Lt 15	Rt 27 Lt 27	Rt 0.22 Lt 0.19	Failed	Failed	No	Reconstruction plate	Failed

**Fig. 2:**
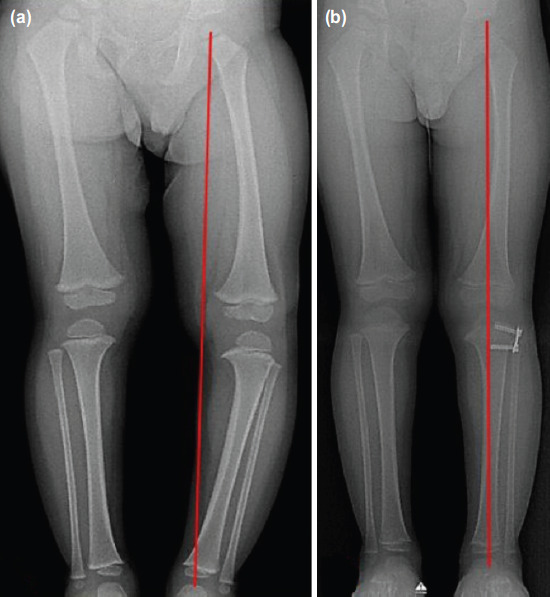
Anteroposterior view scanogram of a 3-year old boy with left tibia vara. The BMI was 25. He was treated with an 8-plate over the proximal tibia (Case 4). (a) The initial mechanical axis (red line) of the left lower limb was in zone 3 (MAD zone 3). (b) Correction was achieved after 14 months (MAD zone 1).

The median pre-operative TFA for genu varus was 20.0° (18.0° to 25.0°) and 20.0° (range 17.0° to 23.0°) for genu valgus. The plates were inserted in the distal femur in three patients (4 knees), and in the proximal tibia in 14 patients (23 knees). There were two patients (3 knees) treated with recon plates and 15 patients (24 knees) used the 8-plates. Fourteen out of the 17 patients achieved complete correction of the deformity (22 out of 27 knees), giving rise to an 81.5% successful rate. An example of a patient with a successful correction is shown in Fig. 2. When analysed according to diagnosis, a successful outcome was seen in all three patients (4 knees) with genu valgus (either idiopathic or Down syndrome) and 81.8% (18 knees) in patients with tibia vara, but this was not significant (p=0.56). A more severe Langeskiold stage (for tibia vara) was associated with a higher failure rate (Table II). Age, pre-operative TFA and duration of treatment did not differ between the successful and failed cases.

**Table II:** Comparison between the successful and failed outcomes of angular deformity correction for different variables. The values for tibia vara, genu valgus and Langeskiold staging are expressed in percentages. Other variables are presented as median and its interquartile range (IQR)

	Outcome		P value
	Resolved N=22	Failed N=5	
Tibia vara (N=23)	81.8%	100%	0.56*
Genu valgus (N=4)			
- idiopathic			
- Down syndrome	18.2%	0	
Langeskiold stage II (N=13)	72.2%	0	<0.05*
Langeskiold stage III (N=8)	27.8%	100%	
Pre-operative TFA (degrees)	20 (16.75-24.0)	20 (20-27)	0.19¥
Pre-removal implant TFA (degrees)	5.5 (5.0-6.25)	19 (12.5-25)	<0.05¥
Duration of treatment (months)	20.5 (13.75-25.5)	27 (24-33)	0.06¥
Correction rate (degrees/month)	0.71 (0.44-1.54)	0.19 (0-0.25)	<0.05¥

*Fisher exact test ¥Mann-Whitney U test

For the 22 knees that were successfully treated, the median rate of correction was 0.71° (0.39-1.55°) per month for proximal tibia, while distal femur procedures revealed a rate of correction of 0.67° (0.61-1.38). The median time required to complete correction was 20 months and duration of implant in situ was 20.5 months (13.75-25.5 months). Spearman’s correlation test showed a strong positive correlation between rate of correction and the pre-operative TFA (rs=0.75, p<0.05). The rate of correction was higher in patients with a larger deformity. However, there was no correlation found between the rate of correction to the age of the child or BMI.

**Fig. 3:**
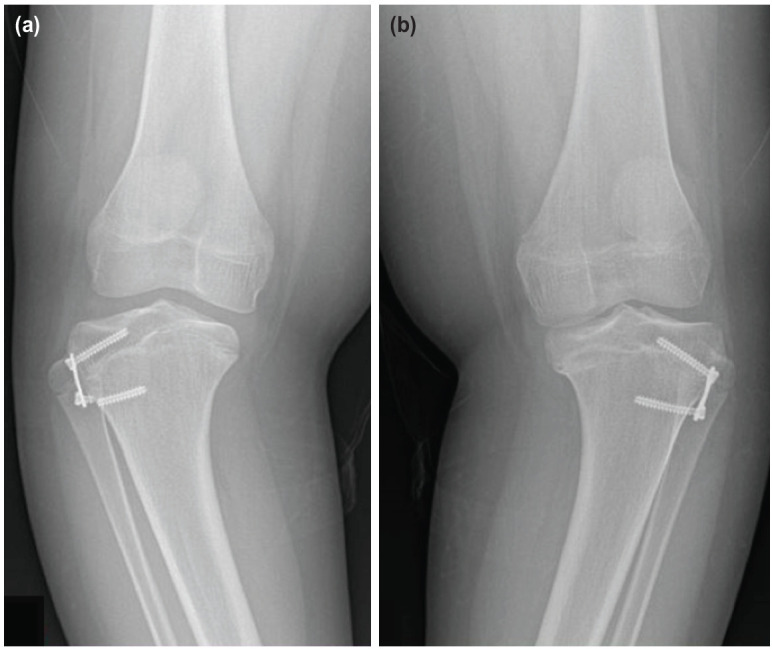
Anteroposterior view knee radiographs of a 16-year old boy with bilateral tibia vara and a BMI of 32. He was treated with 8 plates at the age of 13 years (Case 9). (a) Broken metaphyseal screw in the right tibia. (b) Left tibia metaphyseal screw was bent and subsequently broke prior to removal.

**Fig. 4:**
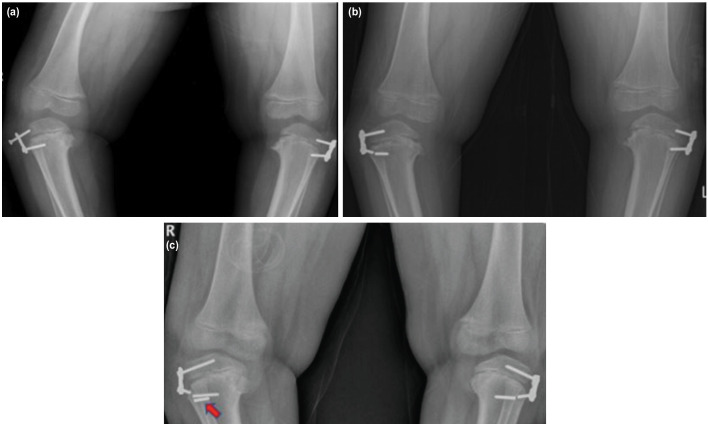
Radiographs of a 3-year-old boy with bilateral tibia vara and a BMI of 28 treated with reconstruction plates (Case 17). (a) Right tibia epiphyseal screw pull-out six months after the primary surgery. Minimal divergence of left tibia screws, which appeared to be short. (b) After revision of the right tibia epiphyseal screw, the metaphyseal screw broke within 12 months. The screw-divergence on the left tibia was minimal. (c) The screws for both tibia were revised to longer ones, but both metaphyseal screws broke within nine months. The red arrow shows the broken end of a screw left from the previous surgery.

There were complications in three patients (Case 9, 13 and 17). All of them failed to achieve full correction, one had a screw pull-out and repeated screw failures while another patient had broken metaphyseal screws. All three patients had a diagnosis of tibia vara, two of them treated with 8-plate and one by reconstruction plate. They were subsequently treated with corrective osteotomy with either the Ilizarov or hexapod external fixator. The two patients with broken metaphyseal screws had either reconstruction plate or 8-plate used for their procedure. Case 9 had 8-plates inserted in both proximal tibia (Fig. 3) and case 17 had reconstruction plates for both tibias (Fig. 4). Case 17 had complications from separate procedures; initially a screw pull-out and then had broken distal metaphyseal screws in two subsequent surgeries. After each revision surgeries, the screws repeatedly failed at the metaphyseal region once it reached the maximal divergence. Cases with failed outcome were associated with Langeskiold stage III, a higher BMI and pre-removal implant TFA but slower rate of correction (Table II).

**Fig. 5:**
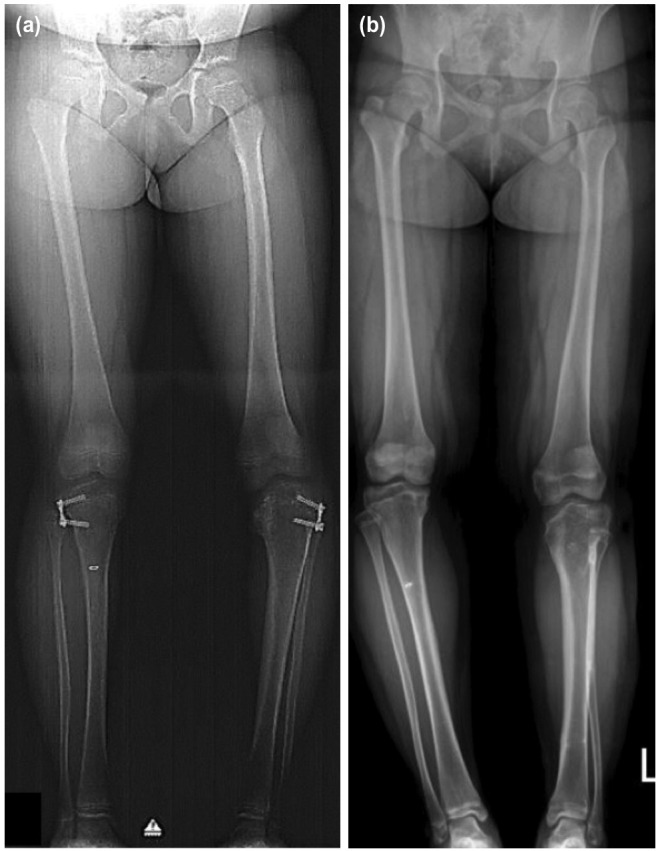
Anteroposterior view scanogram of a 6-year old girl with bilateral tibia vara and a BMI of 26. She was treated with 8-plates (Case 13). (a) Post-operative radiograph showing successful correction of the right tibia, but minimal improvement of the left tibia after 21 months. (b) Rebound deformity to MAD zone 3 on the right tibia five years after implant removal. Corrective osteotomy had been performed on the left tibia.

**Fig. 6:**
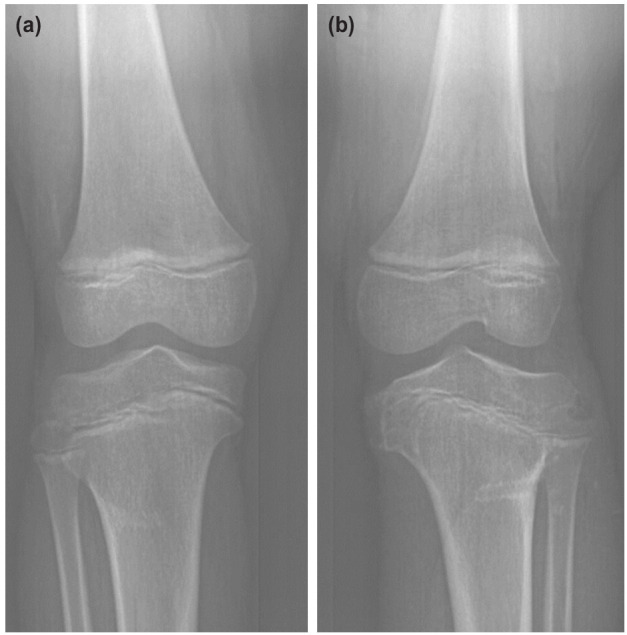
Knee radiographs of a 6-year-old girl who had bilateral 8-plates inserted for tibia vara at the age of 3. The plates were removed after 27 and 38 months for the right and left tibia, respectively. The lateral growth plates appear healthy and there were no signs of growth arrest.

We were able to follow-up five patients for at least 12 months after hardware removal (ranging from 12 to 104 months). Case 13 had his right tibia successfully treated with guided growth after 21 months, but the left tibia did not improve and was subsequently treated with a corrective osteotomy. The right tibia had recurrence of the varus angulation following plate removal (Fig. 5). The patient refused any further surgery. Case 8 had rebound deformity 14 months after removal of hardware. Three more patients (Cases 2, 11 and 12) who were followed-up for more than a year did not show any recurrence of the deformity. Permanent physeal arrest did not occur in our patients after the guided growth procedures (Fig. 6).

## Discussion

Peter Stevens first introduced the technique of guided growth utilising 8-plates in 200611. He described the tension band effect by the plate, rather than compression of the physis exerted by other guided growth methods such as staples and screws. The 8-plate has the advantage of being minimally invasive, reversible, easily inserted and removed while physis and periosteum are spared. In treating patients with various pathologies, this technique was reported to have 30% faster correction than staples and much less complication of implant extrusion^11^.

In 2007, Stevens published a preliminary series that showed successful correction with a low risk of rebound deformity^12^. More reports of the success of the technique were to follow; specifically, in post-traumatic tibia valgus^13^, pathological condition i.e rickets^14^ and also in knee flexion deformities^15^. Other authors also concurred with the effectiveness of the 8-plate in treating angular deformities. Burghardt and Herzenberg reported 93% successful correction of 54 cases treated with the 8-plate, with improvement in both mechanical axis and joint orientation angles of the knee^16^.

Ballal *et al* achieved a mean rate of tibiofemoral angle (TFA) correction of 0.70 per month in the femur, 0.50 per month in the tibia, and 1.20 per month when the femur and tibia were treated concurrently^17^. Another study of 58 patients treated with 8-plates showed restoration of the TFA to within the physiological range in 52 patients (89.7%)^1^. The mean degree of correction was 11 ± 4.9° (range 0° - 25°), and the mean degree of correction per month was 0.93 ± 0.82° (range 0° - 6° per month). More recently, a large series of 967 physes treated with 8-plates in a multicentre study revealed 75% - 80% successful correction with the correction rate of 0.77°/month for the femur and 0.79°/month for the tibia^18^. Our study showed a similar outcome of 81.5% successful correction with the rate of 0.71° /month for tibia but a lower rate for femur (0.68°/month).

Most studies investigating idiopathic conditions revealed predictable outcomes with excellent correction and low complications^19-21^. However, many researchers emphasised on the less favourable outcome of guided growth procedures in pathologic conditions or ‘sick’ physes. Castaneda *et al* treated 62 limbs with temporary hemiepiphyseal stapling and found that the overall mean rate of change for patients with Blount disease was 0° per year, compared to 10° per year for patients with genu varum unrelated to Blount disease^2^.

Although Wiemann *et al* did not find any difference when comparing the rate of correction between stapling and 8-plate in their study, higher complications such as failed correction and screw breakage were evident in the pathologic group^3^. In a different study, Boero *et al* compared the outcome of 8-plate in two group of patients- idiopathic and pathologic (mostly skeletal dysplasia and one Blount’s)^1^. They found slower correction rate which led to one failure in the pathologic group, but no hardware failure or physeal closure were seen. We were unable to make any meaningful comparison in our study as we only had two patients with an idiopathic cause, nevertheless in our cohort all the complications only occurred in the Blount’s patients. Heflin *et al* investigated 17 patients with Blount’s following 8-plate procedures and showed 78% normalisation of mechanical axis, three patients had hardware failure and two with rebound deformity^22^. They concluded that as long as there is no medial physeal bar, treatment with tension band plate is most effective for patients less than four years old.

In our series, we had two patients with hardware failures due to screw breakage. Similar incidences were reported in other studies, ranging from 11% - 44% of the cases^22-24^. A study by Schroerlucke *et al* had eight cases (between the ages of 9 to 12 years) with breakage of the metaphyseal screws on the tibia, all involving Blount patients^23^. Although there was no direct association of the hardware failures with body weight, the combination of heavier patients and abnormal motion at the diseased physis was suggested. Heflin *et al* had a lower incidence in their series and dismissed this complication as minor technical failures^22^. They had three screw failures that occurred only in adolescent subjects, two of them were overweight. Interestingly, one of our patients (Case 9) who had 8-plate screw failures was an adolescent tibia vara with a BMI of 32 and treated at the age of 13 years. Late-onset tibia vara may behave differently than infantile tibia vara and the combination of older age and excessive weight could have contributed to the hardware failures. Further studies should be performed to focus on this age group.

**Fig. 7:**
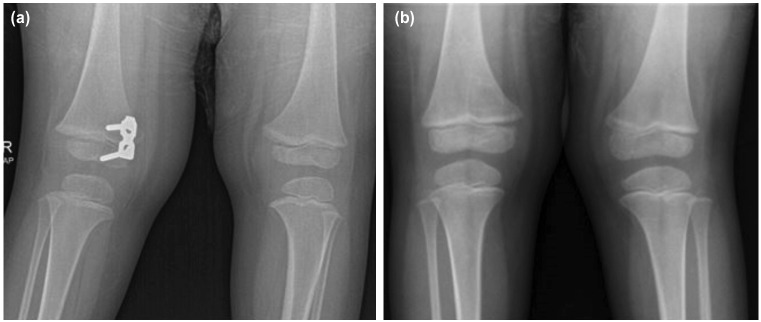
Knee radiographs of a 3-year-old boy with a BMI of 22. He had an idiopathic right genu valgus treated with a reconstruction plate (Case 2). (a) Reconstruction plate inserted at distal medial femur. (b) Correction of angular deformity in the right knee was maintained, 24 months after plate removal.

Some authors suggested that a cannulated titanium screw is biomechanically inferior than a solid stainless-steel screw. Other options would be adding more screws (either two plates or a 4-hole plate) or use non-cannulated and larger core diameter screws^21,24,25^. Lee *et al* had no screw failures in their series of 16 patients treated with non-cannulated plate system and proposed that pre-contouring the plates to ensure coaptation to bony surfaces might have avoided this complication^26^. On a different note, they revealed that cost can be a factor when choosing an implant, as an 8-plate can cost five times more than a reconstruction plate. This also true in our setting. We had two patients who were treated with reconstruction plates; one with idiopathic genu valgus who was successfully corrected (Case 2, Fig. 7) but the other with tibia vara (Case 17, Fig. 4) had screw pull-out and breakages on different occasions. In case 17, we noticed some technical issues whereby the screws were smaller in diameter (3.5mm, compared to 4.5mm in 8-plates cases) and also short in length, which led to the pull-out in the initial surgery. The subsequent screw breakages might be due to the small screw diameter and our failure to position and pre-contour the plates optimally, as suggested by Lee *et al*^26^. Because of 3-point bending, screw failures almost always occur at the metaphyseal area where the shank enters the lateral cortex^24^. In addition, using a 3-hole plate instead of 2-hole in our case could also allow a better plate positioning and a greater arc for optimal screw divergence to avoid this complication. Otherwise, a quad plate or double plate could also be an option. Another technical point is to ensure proper screw tightening technique by alternately tightening the 2 screws for a better plate coaptation to the bone^24^. Other authors used the one-third tubular plates for guided growth. Their results also showed no hardware failures, but the results were preliminary with only eight patients, none of them were Blount^27^.

Rebound deformity is another consequence of guided growth that is difficult to predict. Frequency and amount of overcorrection were variable in the literature. One study suggested there would be a rebound mechanical axis deviation of 1.0mm per month on average^16^. Overcorrection of 3° to 5° was proposed in treating Blount’s with tension band plate^22^. A large series investigating rebound phenomenon following tension band plating reported that younger age and higher degree of deformity (>20°) at implantation are risk factors for a potential recurrence needing revision surgery^28^. However, they cautioned against overcorrecting every patient as not all develop rebound in the high-risk group. In the present study, we were unable to truly analyse this issue due to the short follow-up in many patients. Only five children had long follow-ups after hardware removal (at least 12 months), so we can only assume that the majority of patients did not turn up because they had no recurrence. We have seen two cases of rebound deformity following implant removal in tibia vara. One of the patients (Case 8) had the most severe angulation (37°) in our cohort and was treated at the age of 3. The other is Case 13 who was described earlier (Fig. 5). In contrast, we found two other cases of tibia vara who did not have a recurrence. They had relatively lesser degrees of angulation than the ones who recurred, but otherwise had similar body weights and implants used. All of them had neutral mechanical axis upon removal of hardware, as we do not routinely ‘overcorrect’ in our practice.

Other than the short follow-up, our study was limited by the small number of patients and retrospective in nature. For future improvements we caution the parents about the possibility of a rebound requiring revision surgery especially in the younger child and ensuring follow-up monitoring for more than a year following implant removal to detect recurrence.

## Conclusion

We have shown that our outcome for guided growth to correct knee angular deformity was similar to other studies. Our series revealed that TFA up to 37° can be corrected with rate of correction about 0.7°/month and duration of treatment of around 20 months. Guided growth is safe to perform in children below 12 years old and has good outcome in idiopathic genu valgus and Langeskiold II for tibia vara.
